# Label free technologies: Raman micro-spectroscopy and multi-spectral imaging for lymphocyte classification

**DOI:** 10.1186/1746-1596-8-S1-S32

**Published:** 2013-09-30

**Authors:** Teddy Happillon, Valérie Untereiner, Abdelilah Beljebbar, Cyril Gobinet, Michel Manfait, Sylvie Daliphard, Pascale Cornillet-Lefebvre, Xavier Troussard, Jesus Angulo, Santiago Velasco-Forero, Véronique Saada, Georges Flandrin, Jacques Klossa

**Affiliations:** 1 MEDyC FRE/CNRS 3481, 51096 Reims, France; 2 CHU de Reims, 51100 Reims, France; 3 CHU de Caen, 14033 Caen, France; 4 CMM-ARMINES, 77300 Fontainebleau, France; 5 IGR-Institut Gustave-Roussy, 94805 Villejuif, France; 6 TRIBVN, 39, rue Louveau, 92320 Châtillon, France

## Background

Current diagnostic and prognostic approaches in oncology use morphological and molecular techniques which lead to patient personalized therapies. However, they are still complex and hard to standardize. This is also true for Chronic Lymphocytic Leukemia (CLL) that has been chosen for the IHMO project [1,2]. Simplifying diagnostic processes and making easier the standardization would be highly suitable. In order to develop such a simpler automated method, the IHMO project, funded by the French National Research Agency, proposed to develop a multimodal microscopy scanning platform that includes in a single machine a Raman micro-spectrometer (RMS) combined with a multispectral imager (MSI) [3,4].

RMS is a quick non-invasive and non-destructive technique for tissues and cells analysis [5]. It is very sensible to molecular changes and it could be used as a powerful diagnostic and prognostic tool when used in association with multivariate statistical methods. It is particularly useful for characterizing pathological tumors especially at the cellular level [6,7]. Multispectral imaging in the visible spectrum could confirm RMS classifications and provide new morphometric findings [8].

CLL disease is characterized by the proliferation of lymphocytes (lymphocytosis). It is the most common leukemia, preferentially affecting people aged over 50 years old. It is incurable and in most cases shows no clinical signs. Thus, it is often discovered by chance during a blood test. If necessary, morphological and immunological studies are led by analyzing blood smears colored with May-Grünwald Giemsa, by making a complete blood count and by computing a Matutes score. Such studies are necessary because it is impossible to distinguish a healthy cell from a cancerous one only using a conventional microscope.

## Material and methods

**Figure 1:**
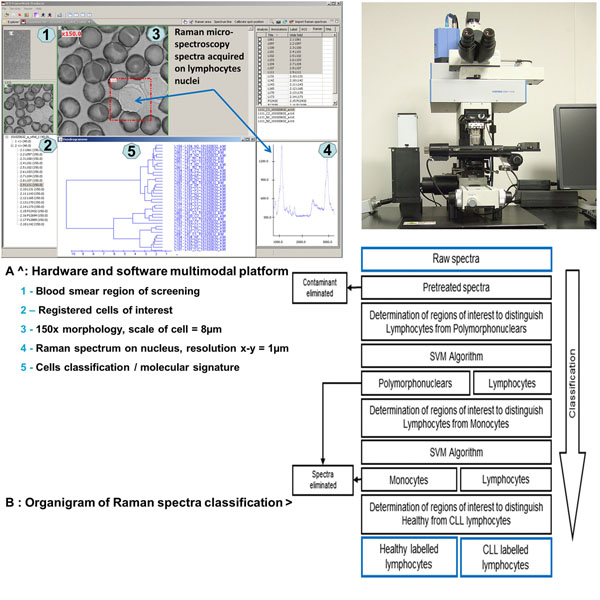
IHMO platform includes the Raman micro-spectrometer and the multi-spectral sources (hardware) and a software able to control the acquisitions and the classification process.

Blood smears were prepared on classical glass slides commonly used in laboratories for microscopy; cells were localized by optical microscopy. A new multimodal machine which has been developed combining i) a 10 bands multi-spectral imager using the full spectrum of transmitted visible light ii) a Raman micro-spectrometer, equipped with a 532nm diode laser excitation source delivering 13.5mW of power on the sample; iii) a microscope stand with 40x and 150x lenses suited with an xyz motorized stage for scanning the blood smear, and localizing x-y coordinates of a representative series (~100 for each patient) of lymphocyte cells before registering Raman spectra on their nuclei and their individual multi-spectral images. An additional piezo actuator allowed for precise z stack recording. Figure 1 shows each step from the screening of the smears to the final results.

### Raman micro-spectroscopy

More precisely, 997 polymorphonuclears, 95 monocytes and 1127 lymphocytes from 12 different blood samples have been considered in the first part of this study, and a Raman spectrum has been acquired on each of them. In the second part, 4257 spectra have been registered on lymphocytes of 49 leukemic patients suffering from hyper leukocytosis Chronic Lymphocytic Leukemia, and 2596 spectra have been recorded on lymphocytes of 27 healthy individuals. These spectra have been then studied, using around 90 cells per blood sample.

Raman data were first pretreated to erase contaminant information into the spectra and then were analyzed using a multivariate statistical method which put forward the relevant information needed to distinguish in a first time lymphocytes from polymorphonuclears. The spectra are then reduced to their relevant information and classified using a Support Vector Machine algorithm [9]. Then this algorithm was used to develop a classification model splitting leukemic and healthy lymphocytes; 3 sets of data were created, the first one, the training set, composed of the spectra from 6 leukemic patients (513 spectra) and 4 healthy individuals (315 spectra) which was used to create different prediction models, the second set, the validation set, composed of spectra from 33 leukemic patients (2820 spectra) and 13 healthy individuals (1106 spectra) was used to select the best model among all previously computed. Finally, the third set, the test set, composed of spectra from 20 different blood samples (2099 spectra) was used to validate the selected classification model in a blind way.

### Multi-spectral imaging

After acquisition of Raman spectra, the slides are stained using a RAL_DIAGNOSTICS™ standardized staining protocol. Then, for each target cell, a multi-z/multi-spectral image is acquired: a z-stack of 100 nm spaced of 24 monochrome images for each of the ten wavelength bands. An algorithm combining mathematical morphology techniques and sparse regression was developed to produce a color RGB image with an extended-depth-of-focus from the multi-z/multispectral image. This color image is used to visually confirm the classification of cells characterized by RMS. The set of scalar images multi-Z/multi-spectral of each cell is also processed by mathematical morphology techniques to produce morpho-spectral texture descriptors reducing the 240 images from each color band to a set of 4 representative images. Such data reduction process allowed representing each cell by a 40x40 square symmetrical correlation matrix. Then, using tools from information geometry and multivariate statistics, cells are embedded into a dimensionality reduced space used to produce an unsupervised classification into two classes: leukemia patients vs. healthy individuals.

## Results and discussion

First stage molecular classification with Raman spectrometry aimed at identifying the nucleated components from a blood smear, mainly polymorphonuclears and lymphocytes. Indeed such differentiation is straightforward with morphological methods like MSI even on unstained samples. Molecular classification gave a sensitivity of 99.3% and a specificity of 99.6%.

The second stage aimed at classifying spectra from leukemic and healthy lymphocytes and provided a sensitivity of 80% and a specificity of 100% on an extended set of blind samples.

It has to be mentioned that in a first step of this work, we tried to match the classification results to the percentages obtained with flow cytometry in term of T, B and NK lymphocytes of a blood sample. However, these results could not be compared with each other since in this study the information which is taken into account is obtained from the nucleus of each white blood cell. As a result, this is a molecular and nuclear information while, in classical cytology, this is the membrane of the cells which is considered.

**Figure 2:**
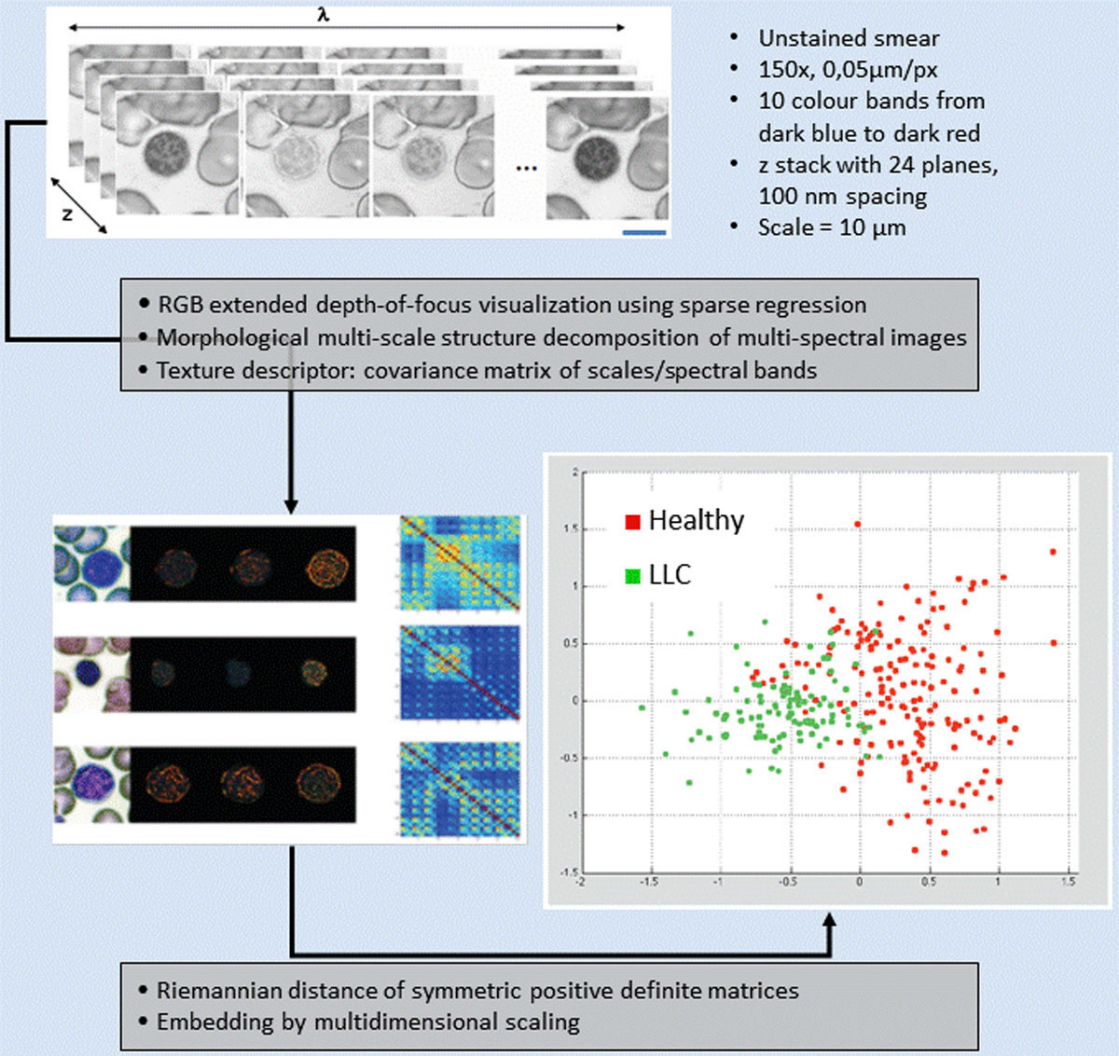
multi-spectral imaging feature extraction and classification method

Morpho-spectral texture classification from multispectral imaging in the visible is used to complement and consolidate the RMS data classifications and in on-going work will be used to identify specific morpho-spectral characteristics associated to leukemic cells (see figure 2).

## Conclusion

IHMO project has demonstrated the power of Raman micro-spectroscopy coupled with supervised classification algorithm such as Support Vector Machines for cell classification and more precisely for the diagnosis of CLL. Morphological descriptors obtained from multi-Z and multispectral images provide another independent classification that still needs to be assessed.

The multimodal microscopy platform can be used more generally in the field of cytohematology, however application to cytological and histological pathology would need further developments and could take profit from new methods in data classification.

## List of abbreviations

RMS: Raman Micro-Spectroscopy; MSI: Multi-Spectral Imaging; CLL: Chronic Lymphoid Leukemia; SVM: Support Vector Machines

## Competing interests

The authors declare that they have no competing interests.

## Authors’ contributions

• TH carried out the acquisitions, pretreatments, the highlighting of relevant information into the spectra, the classification of the data and drafted the extended abstract.

• AB drove the implementation of pretreatments adapted to spectral data and found a way to highlight the relevant information into the spectra.

• VU realized the acquisitions of the data and drafted the extended abstract.

• CG contributes to the choice of the classification algorithm among several studied (supervised or not), and participated in the choice of each possible settings of these algorithms to improve the obtained results.

• JA and SVF developed the multi-spectral classification method and produced the multi-spectral classification.

• XT, JK and MM both designed and managed the full project with the contribution of GF.

• XT, SD, VS and PCL selected the best CLL patients, provided the corresponding samples and patient’s data, and realized the flow cytometry for each of them.
